# Opioid and Nonopioid Analgesic Prescriptions for Dental Visits in the Emergency Department, 2015–2017 National Hospital Ambulatory Medical Care Survey

**DOI:** 10.5888/pcd18.200571

**Published:** 2021-06-10

**Authors:** Shillpa Naavaal, Uma Kelekar, Shital Shah

**Affiliations:** 1 Department of Dental Public Health and Policy, School of Dentistry, Virginia Commonwealth University, Richmond, Virginia; 2 Oral Health Equity Core, Institute for Inclusion, Inquiry and Innovation, Virginia Commonwealth University, Richmond, Virginia; 3 School of Business, College of Business, Innovation, Leadership and Technology, Marymount University, Arlington, Virginia; 4 Department of Health Systems Management, Rush University, Chicago, Illinois; 5 Department of Emergency Medicine, Rush University Medical Center, Chicago, Illinois

## Abstract

**Introduction:**

Prescription and nonprescription opioid misuse and the rising number of dental visits in emergency departments (EDs) are growing public health concerns in the US. Our study objective was to examine the relationship between prescription analgesics (opioids and nonopioids) and the type of ED visits (dental and nondental) at the national level.

**Methods:**

We used data from the 2015–2017 National Hospital Ambulatory Medical Care Survey to examine the association between opioid, nonopioid, and combination of opioid and nonopioid analgesic prescriptions and dental and nondental visits in the ED. Covariates included socioeconomic variables, time of visit, provider type, triage level, hospital location (urban vs rural), and pain level. We conducted descriptive, bivariate, and multivariable analyses using weighted estimates.

**Results:**

The final study sample included 57,098 ED visits from approximately 6 million dental and 414 million nondental visits to EDs during 2015–2017 nationally. Among dental visits, 20.8% received nonopioid analgesics (vs 23.4% among nondental visits), 36.6% received opioid analgesics (vs 14.0% among nondental visits), and 17.7% received both opioids and nonopioid analgesics (vs 8.7% among nondental visits). Adjusted multinomial logistic regression model indicated that, compared with nondental visits, dental visits had 4.8, 1.9, and 3.4 times higher likelihood of receipt of an opioid, nonopioid, or both opioid and nonopioid analgesic prescription, respectively, in the ED than no analgesic prescriptions.

**Conclusion:**

Dental visits resulted in receipt of a significantly higher proportion of opioid prescriptions compared with nondental visits during 2015–2017. The study findings highlight the need for developing interventions to reduce opioid prescriptions in the ED, especially for dental visits.

**Summary**

**What is already known on this topic?**

Unnecessary opioid prescriptions can lead to opioid misuse. Dental visits to an emergency department result in high-cost visits with symptomatic treatment involving antibiotics and analgesics.

**What is added by this report?**

Using the most current national data from emergency departments, we provide estimates of opioid and nonopioid analgesic prescriptions for dental and nondental visits and identify factors associated with analgesic prescriptions in US emergency departments.

**What are the implications for public health practice?**

Study findings highlight the need to reduce the use of opioid prescriptions for dental visits and develop interventions to prevent unnecessary opioid prescriptions in emergency departments.

## Introduction

The number of dental visits to emergency departments (EDs) increased from 2000 to 2014 (1–3). Most dental visits to the ED are nontraumatic, pain-related, and generally a sequel to untreated dental disease that is preventable with routine care (2,4). These dental visits in the ED result in a high rate of prescriptions for antibiotics and analgesics, including opioids, nonopioids, or a combination of both. A study that used 1997–2007 ED data reported a rising trend in prescription drugs for dental visits: almost 74% of dental visits resulted in receipt of analgesic prescription and 54% resulted in receipt of antibiotic prescription (5). Another study that examined dental visits to the ED during 2007–2010 found that 1 in 2 nontraumatic dental visits in the ED resulted in receipt of an opioid prescription (6).

Prescription opioids, used for both acute and chronic pain management, have the highest likelihood of misuse, addiction, and overdose among all prescription drugs (7). Opioids are the leading cause of injury-related deaths in the US. More than 230,000 deaths have been attributed to prescription opioid overdose in the past 2 decades (8). With new policies and programs created for supporting judicious opioid prescribing, the overall opioid prescribing rate in the US has been declining. However, the number of opioid prescriptions per person in 2015 was still 3 times higher than it was in 1999 (9). Nearly half of the patients who entered an opioid abuse treatment program reported first exposure to opioids through a physician’s prescription for pain management, suggesting a high occurrence of prescription opioid misuse (10).

Opioid prescriptions, although common in EDs because of the urgent and pain-related nature of visits, are disproportionately prescribed for some conditions (11). In 2020, Rui and colleagues reported that even though the percentage of ED visits with opioid prescriptions had decreased from 2010–2011 through 2016–2017, dental pain remained one of the top 2 diagnoses for opioid prescriptions in the ED (12). In 2016–2017, 49.7% of dental pain visits resulted in receipt of an opioid prescription, compared with 66.0% in 2010–2011. The current evidence suggests that the combination of ibuprofen and acetaminophen is more effective than opioids in relieving dental pain (13). The use of nonsteroidal anti-inflammatory drugs (NSAIDs) offers a better balance between the benefits and harms of analgesics and optimizes efficacy while minimizing acute adverse events for dental pain–related visits. The American Dental Association also recommends considering NSAIDs as the first-line therapy for acute pain management (14).

In the light of the high number of opioid abuse events and overdose deaths and a rising number of dental visits to the ED, our study objective was to estimate the current prevalence of opioid and nonopioid analgesics prescribed for dental visits and examine the factors associated with the type of analgesic drug prescribed in the ED. We hypothesized that dental visits in the ED would result in receipt of a higher proportion of opioid prescriptions and a lower proportion of nonopioid prescriptions than nondental visits.

## Methods

### Data source

We pooled publicly available and de-identified cross-sectional 2015–2017 National Hospital Ambulatory Medical Care Survey (NHAMCS) data for this study. The NHAMCS is designed to collect data on the utilization and provision of ambulatory care services in hospital emergency and outpatient departments and ambulatory surgery locations in 50 states and the District of Columbia (excluding federal, military, and Veterans Administration hospitals). The survey uses a complex multistage probability design with samples of area primary sampling units (PSU) as first stage, then hospitals within PSUs and all emergency service areas (ESAs) within the EDs, and then ESAs (15). Our study was based on a national sample of 57,098 unweighted ED visits (weighted ED visits, 420,604,880) during the 2015–2017 NHAMCS. The National Center for Health Statistics Ethics Review Board approves the conduct of the NHAMCS. No separate institutional review board approval was required.

### Outcome and predictor variables

Type of analgesic prescription was the primary outcome variable; we classified type as no analgesic, an opioid analgesic, a nonopioid analgesic, or a combination of an opioid and nonopioid analgesic. We identified analgesic prescriptions by searching Multum Lexicon codes. The Multum Lexicon level provides a 3-level nested category system that assigns a therapeutic class to each drug and each ingredient. We identified analgesic prescriptions by using central nervous system agents (level 1 Lexicon code 057) with analgesic therapeutic effects (level 2 Lexicon code 058). Opioid analgesics were classified by using level 3 therapeutic category codes for narcotic analgesics (code 60) and narcotic–analgesic combinations (code 191). The remaining categories of level 3 were classified as nonopioid analgesics (16).

Our primary independent variable of interest was type of ED visit. We identified dental visits by using reason-for-visit variables. The patient could provide up to 5 reasons for a visit. If any of the following codes were stated in the reasons for the visit, the visit was classified as a dental visit: symptoms of teeth and gums (code 1500.0), toothache (code 1500.1), gum pain (code 1500.2), bleeding gums (code 1500.3), dental abscess (code 2675.1), and dental cavities (code 2675.2). We categorized all other visits as nondental visits. We used the reason for the visit instead of discharge diagnosis, because it is more representative of the patient’s perceived problem (17) and may allow more accurate identification of ED dental visits. The use of discharge diagnosis might have introduced a bias and misclassification of a dental visit if a patient incidentally reported a nonurgent dental problem at the time of visit. Also, the transition from ICD-9-CM (*International Classification of Diseases, Ninth Revision, Clinical Modification* [18]) to ICD-10-CM (*International Classification of Diseases, Tenth Revision, Clinical Modification *[19]) codes, which took place in October 2015, could have introduced a bias in dental visit identification as providers started using the new coding system.

Other covariates included pain level, which was categorized on a scale of 0 to 10 (mild, 0–3; moderate, 4–7; severe, 8–10) or as unknown; age in years (<18, 18–44, and ≥45); race/ethnicity (Hispanic, non-Hispanic White, and non-Hispanic Black or “other” [includes Asian, Native Hawaiian/Other Pacific Islander, American Indian/Alaska Native, >1 race]); and payer type. We classified payer type into 4 categories: 1) private insurance, 2) Medicare/other (includes workers’ compensation; other sources of payment, including TRICARE, state and local governments, private charitable organizations, and other liability insurance; and unknown), 3) Medicaid/CHIP (includes Children’s Health Insurance Program [CHIP] and other state-based programs), and 4) self-pay/no insurance (includes no charge or charity/uninsured and self-pay). The time of the visit was categorized as weekday (Monday–Friday) or weekend (Saturday and Sunday). Hospital urban–rural location was categorized by designating metropolitan statistical areas (MSAs) as urban and non-MSA areas as rural. We also categorized location by geographic region (Northeast, Midwest, South, and West). Additional variables were sex (male/female); triage level (urgent, including emergent, immediate, urgent; semi-urgent; nonurgent [including no triage and visit occurred in ESA]; and unknown); and type of physician seen (only a physician seen [included ED attending physician/resident or intern/consulting physician], only an advanced practice provider [APP] seen [included nurse practitioner/physician assistant], both physician and APP seen, and other).

### Statistical analyses

We merged 3 years of NHAMCS ED data; the unit of analysis was visit. All analyses accounted for the complex survey design, and estimates were weighted unless specified otherwise. We made estimates for each variable by using nonmissing data for that variable. We used descriptive statistics to examine the characteristics of all visits and dental and nondental visits. We also examined visit characteristics by the type of analgesic prescribed. We conducted Rao–Scott adjusted χ^2^ tests to test for differences in ED visits and analgesic prescriptions across patient characteristics. We used a multinomial logistic regression model to estimate the relative risk of receiving an opioid analgesic prescription, a nonopioid analgesic prescription, and prescriptions for both opioid and nonopioid medications, compared with no receipt of an analgesic prescription. Using the regression model, we calculated adjusted risk ratios (aRRs), corresponding 95% CIs, and marginal probabilities for each predictor variable. We used Stata version 15 (StataCorp LLC) and α of .05 for all statistical analyses.

## Results

The total number of dental-related visits in the US during 2015-2017 was almost 6 million (unweighted n = 810), which accounted for 1.4% of all ED visits. Nearly 3.6% of dental-related visits were missing information on pain level, 1.5% on payer type, and 3.0% on triage level.

### Visit characteristics and distribution of analgesic prescriptions

Of all ED visits, 55.2% of visits were among females, 76.8% among adults aged 18 or older, and 58.6% among non-Hispanic White people (Table 1). More than one-third (34.4%) of the visits had a report of Medicaid/CHIP as the payer, 84.4% were from urban areas, and 44.2% of visits had a report of moderate or severe pain. More than two-thirds (68.3%) of ED visits were triaged as semi-urgent or urgent, and 73.0% of visits were seen only by a physician.

**Table 1:** Characteristics of Emergency Department Visits, National Hospital Ambulatory Medical Care Survey, 2015–2017^a^

Characteristic	Unweighted n	All Visits (Unweighted n = 57,098)	Dental Visits (Unweighted n = 810)	Nondental Visits (Unweighted n = 56,288)	*P* Value^b^
**Weighted no. of visits**	—	420,604,880	5,953,081	414,651,799	—
**Age group, y**					
<18	12,781	23.2 (21.1–25.4)	8.1 (5.7–11.3)	23.4 (21.3–25.7)	<.001
18–44	22,442	38.8 (37.6–40.0)	70.7 (65.8–75.1)	38.3 (37.1–39.6)	
≥45	21,875	38.0 (36.6–39.5)	21.2 (17.9–25.0)	38.3 (36.8–39.8)	
**Sex**					
Male	25,835	44.8 (44.1–45.5)	50.3 (45.8–54.8)	44.7 (44.0–45.4)	.02
Female	31,263	55.2 (54.5–55.9)	49.7 (45.2–54.2)	55.3 (54.6–56.0)	
**Ethnicity/race**					
Non-Hispanic White	33,258	58.6 (55.4–61.7)	63.4 (57.2–69.1)	58.5 (55.3–61.6)	.006
Non-Hispanic Black and “other”^c^	15,175	25.7 (22.6–29.0)	26.5 (20.9–33.0)	25.6 (22.6–29.0)	
Hispanic	8,665	15.8 (13.6–18.1)	10.1 (7.1–14.1)	15.8 (13.7–18.2)	
**Region**					
Northeast	10,037	15.8 (13.2–18.8)	11.4 (8.3–15.4)	15.9 (13.3–18.8)	<.001
Midwest	14,273	24.1 (20.2–28.5)	30.5 (23.2–38.8)	24.0 (20.1–28.4)	
South	20,214	39.0 (34.0–44.2)	44.0 (35.3–53.1)	38.9 (34.0–44.1)	
West	12,574	21.1 (17.7–25.0)	14.2 (10.5–18.9)	21.2 (17.7–25.1)	
**Payer**					
Private insurance	15,471	25.9 (24.3–27.6)	22.0 (18.7–25.7)	26.0 (24.4–27.7)	<.001
Medicaid/CHIP	16,179	34.4 (32.0–37.0)	38.2 (32.8–44.0)	34.4 (31.9–37.0)	
Medicare/other^d^	19,751	30.7 (28.1–33.3)	20.0 (15.1–26.3)	30.8 (28.2–33.5)	
Self-pay/no insurance	4,851	9.0 (7.7–10.4)	19.7 (16.1–23.8)	8.8 (7.6–10.2)	
**Pain scale**					
Mild	14,555	24.8 (22.4–27.3)	11.4 (7.3–17.3)	25.0 (22.6–27.5)	<.001
Moderate	11,474	20.5 (18.9–22.3)	19.4 (15.8–23.6)	20.6 (18.9–22.3)	
Severe	12,945	23.7 (21.5–26.1)	47.7 (40.7–54.8)	23.4 (21.2–25.8)	
Unknown	16,158	30.9 (26.0–36.4)	21.5 (15.3–29.3)	31.1 (26.1–36.5)	
**Time of week seen**					
Weekend	15,344	27.0 (26.6–27.5)	30.9 (26.6–35.5)	27.0 (26.5–27.4)	.08
Weekday	41,754	73.0 (72.5–73.4)	69.1 (64.5–73.4)	73.0 (72.6–73.5)	
**Type of health care provider seen**					
Physician only	42,963	73.0 (70.1–75.6)	61.1 (54.7–67.1)	73.1 (70.3–75.8)	<.001
APP^e^ only	4,824	10.6 (8.7–12.9)	21.9 (16.4–28.6)	10.4 (8.5–12.7)	
APP^e^ and physician	7,678	13.6 (11.4–16.1)	13.5 (9.2–19.4)	13.6 (11.4–16.0)	
Other	1,633	2.9 (2.5–3.3)	3.5 (2.1–5.8)^f^	2.9 (2.5–3.3)	
**Hospital location**					
Urban	49,002	84.4 (75.4–90.5)	77.9 (64.2–87.4)	84.5 (75.5–90.6)	.004
Rural	8.096	15.6 (9.5–24.6)	22.1 (12.5–35.8)	15.5 (9.4–24.5)	
**Triage level^g^**					
Urgent	23,980	42.6 (38.1–47.2)	11.6 (8.9–14.9)	43.0 (38.6–47.6)	<.001
Semi-urgent	14,524	25.7 (22.9–28.6)	44.4 (36.7–52.3)	25.4 (22.7–28.3)	
Nonurgent	6,329	10.1 (7.8–12.8)	20.7 (17.2–24.6)	9.9 (7.7–12.7)	
Unknown	10,842	21.7 (16.4–28.1)	23.4 (16.0–32.9)	21.7(16.4–28.1)	

Abbreviations: APP, advanced practice provider; CHIP, Children’s Health Insurance Program.

a All values are weighted percentage (95% CI) unless otherwise indicated.

b *P* value is from Rao–Scott χ^2^ test.

c “Other” race/ethnicity includes Asian, Native Hawaiian/Other Pacific Islander, American Indian/Alaska Native, and >1 race.

d Other payer includes other sources of payment: workers’ compensation insurance, unknown payer, and other (TRICARE, state and local governments, private charitable organizations, and other liability insurance).

e Nurse practitioner or physician assistant.

f Unreliable estimate because of small sample size (<30) or relative SE > 30%.

g Urgent includes immediate, emergent, or urgent; nonurgent includes no triage and visit occurred in emergency service area.

The proportion of dental visits was larger than the proportion of nondental visits among adults aged 18 to 44, males, non-Hispanic White people, and people with Medicaid/CHIP, living in the Midwest or South, and living in rural areas. On the pain scale, 47.7% of dental visits and 23.4% of nondental visits reported severe pain. Most dental visits were triaged as semi-urgent (44.4%), followed by nonurgent (20.7%), whereas most nondental visits were triaged as urgent (43.0%). By type of provider seen, APPs saw a higher proportion of dental visits than nondental visits (21.9% vs 10.4%).

Overall, 14.3% of ED visits received opioid analgesics only, 23.4% nonopioid analgesics only, 8.8% both opioid and nonopioid analgesics, and 53.4% no analgesic prescription (Table 2). We found a significant difference in opioid prescriptions by type of visit. Among dental visits, 20.8% received nonopioid analgesics (vs 23.4% among nondental visits), 36.6% received opioid analgesics (vs 14.0% among nondental visits), and 17.7% received both opioids and nonopioid analgesics compared (vs 8.7% among nondental visits).

**Table 2:** Distribution of Analgesic Prescriptions by Visit Characteristics for All Emergency Department Visits, National Hospital Ambulatory Medical Care Survey, 2015–2017^a^

Characteristic	No Analgesic (Unweighted n = 30,937)	Nonopioid Analgesic Only (Unweighted n = 13,260)	Opioid Only (Unweighted n = 7,953)	Both Opioid and Nonopioid Analgesics (Unweighted n = 4,948)	*P* Value^b^
**Weighted n**	224,669,832	60,312,077	98,419,241	37,203,730	—
**Total**	53.4 (52.0–54.8)	23.4 (22.4–24.4)	14.3 (13.6–15.1)	8.8 (8.3–9.5)	—
**Type of visit**					
Dental	24.8 (20.2–30.1)	20.8 (17.0–25.3)	36.6 (32.4–41.0)	17.7 (14.4–21.7)	<.001
Non-dental	53.8 (52.4–55.2)	23.4 (22.4–24.5)	14.0 (13.3–14.8)	8.7 (8.1–9.3)	
**Age group, y**					
<18	60.1 (57.8–62.3)	35.1 (33.1–37.1)	2.6 (2.1–3.2)	2.3 (1.8–2.8)	<.001
18–44	49.6 (48.2–51.0)	22.4 (21.3–23.5)	16.6 (15.8–17.6)	11.4 (10.6–12.2)	
≥45	53.2 (51.3–55.2)	17.3 (16.4–18.3)	19.1 (18.0–20.3)	10.3 (9.5–11.2)	
**Sex**					
Male	55.0 (53.5–56.5)	23.1 (22.0–24.3)	13.5 (12.7–14.4)	8.3 (7.7–8.9)	<.001
Female	52.1 (50.7–53.5)	23.6 (22.5–24.7)	15.0 (14.2–15.9)	9.3 (8.6–9.9)	
**Ethnicity/race**					
Non-Hispanic White	53.5 (51.9–55.1)	21.3 (20.2–22.4)	15.9 (15.1–16.9)	9.2 (8.5–10.0)	<.001
Non-Hispanic Black and “other”^c^	54.4 (52.1–56.7)	24.9 (23.3–26.7)	12.3 (11.4–13.3)	8.3 (7.5–9.1)	
Hispanic	51.5 (49.7–53.2)	28.6 (26.6–30.8)	11.6 (10.4–13.0)	8.2 (7.3–9.3)	
**Region**					
Northeast	55.0 (52.8–57.2)	27.7 (25.5–30.0)	9.1 (7.8–10.7)	8.1 (7.2–9.2)	<.001
Midwest	52.0 (49.4–54.6)	25.2 (23.1–27.3)	13.9 (12.3–15.8)	8.9 (8.1–9.7)	
South	53.4 (50.7–56.0)	21.8 (20.2–23.5)	16.0 (14.7–17.3)	8.8 (7.7–10.1)	
West	53.9 (51.5–56.2)	21.1 (19.1–23.3)	15.6 (13.8–17.7)	9.4 (8.1–10.8)	
**Payer**					
Private insurance	51.6 (49.8–53.5)	22.7 (21.5–23.8)	15.3 (14.3–16.4)	10.4 (9.5–11.4)	<.001
Medicaid/CHIP	55.5 (53.4–57.6)	19.2 (17.7–20.8)	16.9 (15.6–18.5)	8.4 (7.5–9.3)	
Medicare/other^d^	53.9 (52.4–55.4)	27.9 (26.5–29.4)	10.5 (9.7–11.5)	7.6 (7.0–8.3)	
Self-pay/no insurance	49.8 (47.1–52.6)	22.5 (19.9–25.3)	16.9 (15.6–18.5)	10.7 (9.4–12.1)	
**Pain scale**					
Mild	72.1 (70.1–74.1)	19.4 (18.1–20.9)	5.2 (4.5–6.1)	3.2 (2.6–3.8)	<.001
Moderate	45.2 (43.6–46.8)	27.9 (26.4–29.6)	16.2 (15.1–17.3)	10.7 (9.8–11.6)	
Severe	29.4 (27.6–31.1)	25.0 (23.8–26.3)	27.9 (26.4–29.4)	17.7 (16.5–19.1)	
Unknown	60.8 (58.9–62.3)	22.8 (20.9–24.8)	10.6 (9.7–11.7)	5.8 (5.2–6.4)	
**Time of week seen**					
Weekend	53.3 (51.4–55.1)	23.9 (22.4–25.4)	14.2 (13.1–15.3)	8.7 (7.9–9.5)	.62
Weekday	53.5 (52.1–54.8)	23.2 (22.3–24.2)	14.4 (13.6–15.2)	8.9 (8.3–9.5)	
**Type of health care provider seen**					
Physician only	53.6 (52.0–55.1)	22.9 (21.8–24.0)	14.9 (14.1–15.8)	8.7 (7.9–9.4)	<.001
APP^e^ only	49.6 (46.1–53.2)	28.8 (25.6–32.2)	12.7 (10.9–14.8)	8.9 (7.6–10.4)	
APP^e^ and physician	48.4 (45.9–50.8)	25.3 (23.4–27.3)	15.1 (13.6–16.6)	11.3 (10.1–12.6)	
Other	87.7 (84.3–90.5)	7.6 (5.8–9.9)	3.1 (2.0–4.7)	1.6 (0.9–2.7)^f^	
**Hospital location**					
Urban	52.8 (51.3–54.3)	23.6 (22.5–24.8)	14.6 (13.7–15.4)	8.9 (8.3–9.7)	.13
Rural	56.6 (53.5–59.7)	22.1 (19.3–25.2)	13.1 (11.4–15.1)	8.1 (7.1–9.3)	
**Triage level^g^**					
Urgent	51.6 (50.2–53.1)	22.2 (21.1–23.4)	16.0 (15.1–16.9)	10.1 (9.3–11.0)	<.001
Semi-urgent	49.2 (47.3–51.1)	28.7 (26.9–30.5)	13.1 (12.1–14.1)	9.1 (8.3–9.9)	
Nonurgent	62.2 (59.5–64.8)	19.9 (18.2–21.8)	11.2 (9.9–12.7)	6.7 (5.7–7.8)	
Unknown	56.1 (53.4–58.8)	21.9 (19.6–24.4)	14.5 (13.0–16.3)	7.4 (6.6–8.3)	

Abbreviations: APP, advanced practice provider; CHIP, Children’s Health Insurance Program.

a All values are weighted percentage (95% CI) unless otherwise indicated.

b *P* value is from Rao–Scott χ^2^ test.

c “Other” race/ethnicity includes Asian, Native Hawaiian/Other Pacific Islander, American Indian/Alaska Native, and >1 race.

d Other payer includes other sources of payment: workers’ compensation insurance, unknown payer, and other (TRICARE, state and local governments, private charitable organizations, and other liability insurance).

e Nurse practitioner or physician assistant.

f Unreliable estimate because of small sample size (<30) or relative SE > 30%.

g Urgent includes immediate, emergent, or urgent; nonurgent includes no triage and visit occurred in emergency service area.

During 2015–2017, visits among adults aged 18 or older, females, and people who were non-Hispanic White received a higher percentage of opioids and opioid combinations than nonopioid analgesics. In contrast, visits among non-Hispanic Black/other people (24.9%) or Hispanic (28.6%) people received a higher percentage of nonopioids than any opioid and opioid combinations. By geography, visits in the South and West received a higher percentage of opioids and opioid combinations (24.8% and 25.0%) than visits in the Northeast (17.2%). By payer, visits among people who self-paid or were without insurance (27.6%) received the highest proportion of opioids or opioid combinations, followed by private insurance visits (25.7%). Visits paid by Medicare/other had the lowest percentage of opioid or opioid combinations (18.1%) and the highest percentage of nonopioid analgesics. Pain level and triage level had a dose–response relationship with the receipt of analgesic prescriptions. Visits that reported severe pain and were triaged as urgent received a higher proportion of opioids or opioid combinations than nonopioid analgesics. Compared with visits that were seen by a physician or an APP only, those seen by both a physician and an APP received a higher proportion of opioids or opioid combinations.

### Multinomial logistic regression

Dental ED visits had a significantly higher likelihood than nondental visits of receiving an opioid prescription (aRR = 4.76; 95% CI, 3.53–6.41) than no analgesic after controlling for demographic and clinical characteristics, insurance status, pain scores, and other covariates. Similarly, compared with nondental visits, dental visits were almost twice (aRR = 1.87, 95% CI, 1.31–2.67) as likely to receive a nonopioid analgesic versus no analgesic and 3.4 (aRR = 3.44; 95% CI, 2.39–4.96) times as likely to receive both opioid and nonopioid analgesics versus no analgesic (Table 3). The percentage of dental visits that received an opioid analgesic was nearly 18 percentage points higher than the percentage of nondental visits that received an opioid analgesic (28.4% vs 10.7%) (Figure). Consistently, the probability of receiving both opioid and nonopioid analgesic for a dental visit (13.6%) was twice the probability of being prescribed both an opioid and a nonopioid analgesic for a nondental visit (7.1%).

**Table 3:** Multinomial Logistic Regression Model Comparing Opioid Analgesic, Nonopioid Analgesic, and a Combination of Opioid and Nonopioid Analgesic Prescriptions, With No Opioid Analgesic Prescriptions, National Hospital Ambulatory Medical Care Survey, 2015–2017^a^

Variables	Nonopioid Analgesic Only^b^	*P* Value	Opioid Analgesic Only^b^	*P* Value	Opioid and Nonopioid Analgesic^b^	*P* Value
**Type of visit**						
Nondental visit	Reference		Reference		Reference	
Dental Visit	1.87 (1.31–2.67)	.001	4.76 (3.53–6.41)	<.001	3.44 (2.39–4.96)	<.001
**Pain scale**						
Mild	Reference		Reference		Reference	
Moderate	2.39 (2.17–2.63)	<.001	4.67 (3.89–5.62)	<.001	4.79 (3.98–5.79)	<.001
Severe	3.43 (3.09–3.81)	<.001	11.87 (9.71–14.51)	<.001	12.10 (10.01–14.64)	<.001
Unknown	1.38 (1.24–1.54)	<.001	2.70 (2.18–3.35)	<.001	2.47 (1.98–3.07)	<.001
**Age group, y**						
18–44	Reference		Reference		Reference	
<18	1.50 (1.35–1.67)	<.001	0.19 (0.15–0.23)	<.001	0.23 (0.18–0.30)	<.001
≥45	0.84 (0.77–0.91)	<.001	1.25 (1.13–1.37)	<.001	1.05 (0.94–1.18)	.38
**Sex**						
Female	Reference		Reference		Reference	
Male	0.94 (0.88–1.01)	.11	0.99 (0.93–1.05)	.75	1.01 (0.94–1.09)	.76
**Ethnicity/race**						
Non-Hispanic White	Reference		Reference		Reference	
Non-Hispanic Black and “other”^c^	1.07 (0.98–1.17)	.13	0.74 (0.68–0.82)	<.001	0.87 (0.75–0.99)	.04
Hispanic	1.23 (1.10–1.38)	<.001	0.93 (0.81–1.07)	.31	1.06 (0.93–1.19)	.38
**Payer**						
Private insurance	Reference		Reference		Reference	
Medicare/other^d^	0.91 (0.82–1.01)	.08	0.87 (0.79–0.96)	.006	0.68 (0.60–0.78)	<.001
Medicaid/CHIP	1.07 (0.98–1.16)	.12	0.84 (0.74–0.95)	.006	0.84 (0.75–0.94)	.002
Self-pay/no charge	1.01 (0.86–1.18)	.93	0.91 (0.80–1.04)	.16	0.86 (0.73–1.01)	.07
**Triage level^e^**						
Semi-urgent	Reference		Reference		Reference	
Urgent	0.85 (0.78–0.93)	<.001	1.08 (0.99–1.18)	.10	1.04 (0.91–1.19)	.55
Nonurgent	0.64 (0.55–0.73)	<.001	0.80 (0.69–0.94)	.005	0.72 (0.58–0.89)	.003
Unknown	0.85 (0.72–1.01)	.06	1.07 (0.88–1.30)	.50	0.89 (0.72–1.09)	.26
**Region**						
Northeast	Reference		Reference		Reference	
Midwest	0.94 (0.80–1.11)	.49	1.74 (1.46–2.08)	<.001	1.23 (0.99–1.54)	.06
South	0.78 (0.67–0.90)	.001	1.98 (1.67–2.35)	<.001	1.23 (0.99–1.52)	.06
West	0.70 (0.60–0.81)	<.001	1.71 (1.43–2.03)	<.001	1.19 (0.94–1.5)	.15
**Type of health care provider seen**						
Physician only	Reference		Reference		Reference	
APP^f^ only	1.22 (1.02–1.45)	.02	0.97 (0.83–1.15)	.76	1.11 (0.89–1.37)	.36
Physician and APP	1.17 (1.02–1.34)	.02	1.11 (0.99–1.25)	.07	1.32 (1.15–1.52)	<.001
Other	0.21 (0.15–0.28)	<.001	0.11 (0.07–0.18)	<.001	0.10 (0.06–0.18)^g^	<.001
**Time of week seen**						
Weekday	Reference		Reference		Reference	
Weekend	1.01 (0.94–1.08)	.72	1.00 (0.91–1.09)	.94	0.99 (0.90–1.08)	.81
**Hospital location**						
Urban	Reference		Reference		Reference	
Rural	0.90 (0.72–1.13)	.37	0.81 (0.67–0.98)	.03	0.84 (0.64–1.09)	.19

Abbreviations: APP, advanced practice provider; CHIP, Children’s Health Insurance Program.

a All values are adjusted rate ratio (95% CI) unless otherwise indicated.

b Reference group is “no analgesic prescription.”

c “Other” race/ethnicity includes Asian, Native Hawaiian/Other Pacific Islander, American Indian/Alaska Native, and >1 race.

d Other payer includes other sources of payment: workers’ compensation insurance, unknown payer, and other (TRICARE, state and local governments, private charitable organizations, and other liability insurance).

e Urgent includes immediate, emergent, or urgent; nonurgent includes no triage and visit occurred in emergency service area.

f Nurse practitioner or physician assistant.

g Unreliable estimate because of small sample size (<30) or relative SE > 30%.

**Figure:**
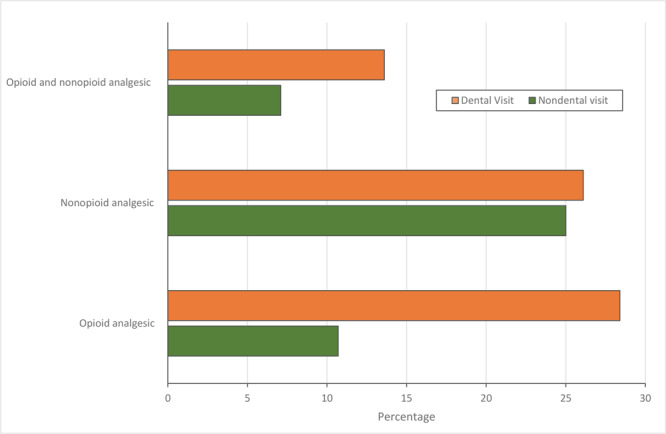
Adjusted marginal probabilities of receiving analgesic prescriptions in emergency departments by type of visit (dental vs nondental), 2015–2017 National Hospital Ambulatory Medical Care Survey. Marginal probabilities were obtained from the regression model adjusted for all included variables.

**Table:** 

Type of visit	Opioid analgesic	Nonopioid analgesic	Opioid and nonopioid analgesic
Nondental visit	10.7	25.0	7.1
Dental visit	28.4	26.1	13.6

Pain was independently associated with analgesic prescriptions. Visits among people with severe pain were approximately 12 times as likely as visits among people with mild pain to receive opioids (aRR = 11.87; 95% CI, 9.71–14.51) or both opioid and nonopioid analgesics (aRR = 12.10; 95% CI, 10.01–14.64). All variables except time of visit and sex were associated with the receipt of analgesic prescriptions. Compared with the visits among adults aged 18 to 44, visits among adults age 45 or older were 25% more likely (aRR = 1.25; 95% CI, 1.13–1.37) to receive an opioid prescription, and visits among people aged 18 years or younger were 50% more likely (aRR = 1.50; 95% CI, 1.35–1.67) to receive a nonopioid analgesic. Compared with visits among people who were non-Hispanic White, visits among non-Hispanic Black/other people were less likely (aRR = 0.74; 95% CI, 0.68–0.82) to receive an opioid prescription and visits among Hispanic people were more likely to receive a nonopioid analgesic prescription (aRR = 1.23; 95% CI, 1.10–1.38). Visits covered by Medicare/other (aRR = 0.87; 95% CI, 0.79–0.96) and Medicaid/CHIP (aRR = 0.84; 95% CI, 0.74–0.95) had a lower likelihood of receiving opioids or receiving both opioid and nonopioid analgesics than visits covered by private insurance.

ED visits in the South (aRR = 1.98, 95% CI, 1.67–2.35), Midwest (aRR = 1.74; 95% CI, 1.46–2.08), and West (aRR = 1.71; 95% CI, 1.43–2.03) had more than 1.7 times higher likelihood of receiving an opioid prescription compared with ED visits in the Northeast. Triage level and type of provider variables were also associated with analgesic prescription, but these data did not have a consistent pattern. Prescription of a nonopioid analgesics (aRR = 1.17; 95% CI, 1.02–1.34) and both opioid and nonopioid analgesics (aRR = 1.32; 95% CI, 1.15–1.52) was more likely in visits that were seen by a physician and an APP than in visits seen by a physician only. However, the likelihood of receiving only an opioid prescription from a physician alone did not significantly differ from the likelihood of receiving only an opioid prescription from an APP alone. Nonurgent and other visits (aRR = 0.81; 95% CI, 0.69–0.94) were less likely to receive opioid analgesics compared with semi-urgent visits.

## Discussion

During 2015–2017, three in 4 dental ED visits resulted in at least 1 analgesic prescription, and more than three-fourths of those prescriptions were for opioid analgesics. On the other hand, although almost half of the nondental visits received any analgesic prescriptions, less than half of those were opioid prescriptions. In the adjusted analyses, we found that dental visits received a significantly higher proportion of opioid prescriptions in the ED than nondental visits. Other factors such as pain scale, age, race/ethnicity, geographic region, urban–rural location, payer type, triage level, and type of provider were also significantly associated with opioid prescriptions in the ED.

Our findings suggest that even after controlling for other factors, dental visits were almost 5 times more likely to receive an opioid, 3 times more likely to receive an opioid and nonopioid combination, and almost 2 times more likely to receive a nonopioid analgesic prescription in the ED compared with nondental visits. This finding is concerning given the relationship between opioid exposure and increased risk of long-term opioid use and abuse among people exposed (20). A recent study found that opioid prescriptions in the ED for dental visits were associated with an increased likelihood of persistent or high-risk opioid use (21). To further explore opioid prescriptions, we analyzed the timing of opioid analgesic prescription (in the ED, at discharge, or at both times) and found that dental visits received a higher proportion of opioids at discharge (43.8%) than nondental visits (25.5%). This finding, combined with our main findings, further highlights that dental visits receive a disproportionate number of opioid prescriptions during discharge, which may increase the potential for opioid misuse.

One explanation for the high proportion of opioid prescriptions for dental visits in the ED could be that most EDs do not have a dental provider on-site or the dental set-up to treat the underlying reason for the dental visit (22). Because most dental visits are pain-related, patients are given analgesics, including opioids, for symptomatic treatment and temporary relief (11). Another reason could be that although ED physicians are trained to treat emergency and acute conditions, they generally receive little training or continuing education on oral health and related emergencies and related pain prescription guidelines (14,23).

Our findings on opioid prescriptions concur with previous research and show that analgesic prescriptions for dental visits, especially opioid prescriptions, are as high as they were a decade ago. Okunseri et al, using 1997–2000 and 2003–2007 data, reported that the prescription rates for nontraumatic dental ED visits were (as an average for both periods combined) 43% for opioid analgesics, 20% for nonopioid analgesics, and 12% for opioid and nonopioid analgesic combinations (24). With 2015–2017 data, we observed a similar proportion of dental visits receiving any analgesics (75.2%). Our findings suggest that the analgesic prescription distribution in 2015–2017 shifted slightly; nearly 36.6% of dental visits received opioids, 20.8% received nonopioid analgesics, and 17.7% received opioids and nonopioid analgesics, but the total percentage of prescriptions for opioid and nonopioid combinations did not change much. The proportion of dental visits receiving opioid and nonopioid combination prescriptions was 54.3% in 2015–2017 compared with 50.3% in 2007–2010 (6).

In addition to type of visit, we found that pain level and age group were the other 2 significant factors associated with receiving an opioid prescription and were consistent with previous findings (6,11). Similarly, visits among non-Hispanic White patients, patients covered by private insurance, and patients in the South, Midwest, and West received a higher proportion of opioid prescriptions than visits among their counterparts (25–27). Opioid prescriptions in the ED can be related to biases in pain management among health care providers and a desire to increase patient satisfaction (28,29), which may explain some of our findings. Our findings suggest that visits attended by an APP and a physician had a higher likelihood of receiving opioid and nonopioid combination or only nonopioid analgesics compared with visits attended by a physician only. A study found that APPs are less likely than physicians to prescribe opioids, which may partly explain this finding (30).

Our study has implications for health care providers, oral health, and public health partners at national and state levels. Because of limited dental care coverage for low-income adults and barriers to dental care access, EDs have become a usual source of care and the number of dental-related ED visits has risen in recent years (1). Providing accessible and affordable dental care for patients across all age groups and income levels can reduce the prevalence and incidence of dental disease and the number of visits to ED, potentially reducing the number and proportion of opioid prescriptions.

Providing opportunities for continuing education in oral health, discussing best practices for pain management for nontraumatic conditions such as dental pain, and promoting programs such as Alternative to Opioid Therapy (ALTO) (31) and the Centers for Disease Control and Prevention’s interactive training series for ED health care providers (32), are some ways to reduce unnecessary opioid prescriptions in the ED (23,33). State-level opioid policies and programs such as prescription drug monitoring programs and continuing medical education requirements for licensure can include information on dental visits and pain management options in the ED to improve awareness and compliance (34). System-level interventions such as pre-populated scripts and flags to guide the use of recommended analgesic medication for a given condition could further help reduce unnecessary opioid prescriptions (33). Lastly, where possible, ED support staff members and social workers could assist with scheduling appointments for ED dental patients and divert them to dental clinics, which could facilitate regular dental care for patients and reduce unnecessary analgesic prescriptions (35).

Our study has several limitations. The NHAMCS consists of visit-level data and does not provide person-level estimates, and as such, we could not identify revisits. However, it is a comprehensive ED data set that provides national estimates. Although NHAMCS provides data on opioids prescriptions, it does not have information on how many prescriptions were filled and taken. Lastly, we did not examine the types of opioids or the number of pills and dosage of opioids prescribed. Nonetheless, our study provides up-to-date estimates for analgesic prescriptions in the ED and identifies associated factors.

Dental visits in the ED receive a disproportionately high number of opioid analgesics. Although opioid prescriptions, in general, have declined since 2012 (8), the rate of opioid prescriptions for dental visits remains high. Our findings urge health care providers, policy makers, and oral health partners to develop interventions to reduce the rate of opioid prescriptions resulting from dental visits to the ED.
